# Author Correction: A natural mouse model reveals genetic determinants of systemic capillary leak syndrome (Clarkson disease)

**DOI:** 10.1038/s42003-019-0691-0

**Published:** 2019-11-25

**Authors:** Abbas Raza, Zhihui Xie, Eunice C. Chan, Wei-Sheng Chen, Linda M. Scott, A. Robin Eisch, Dimitry N. Krementsov, Helene F. Rosenberg, Samir M. Parikh, Elizabeth P. Blankenhorn, Cory Teuscher, Kirk M. Druey

**Affiliations:** 1 Departments of Medicine and Pathology, University of Vermont School of Medicine, Burlington, VT 05405 USA; 2 Lung and Vascular Inflammation Section, Laboratory of Allergic Diseases, National Institute of Allergy and Infectious Diseases, NIH, Bethesda, MD 20892 USA; 3 Department of Biomedical and Health Sciences, University of Vermont School of Medicine, Burlington, VT 05405 USA; 4 Inflammation Immunobiology Section, Laboratory of Allergic Diseases, National Institute of Allergy and Infectious Diseases, NIH, Bethesda, MD 20892 USA; 5 Division of Nephrology and Department of Medicine, Beth Israel Deaconess Medical Center and Harvard Medical School, Boston, MA 02215 USA; 6 Department of Microbiology and Immunology, Drexel University College of Medicine, Philadelphia, PA 19129 USA

**Keywords:** Genetic linkage study, Genetic association study

Correction to: *Communications Biology* 10.1038/s42003-019-0647-4, published online 31 October 2019.

In the original version of this Article, the trait “Histamine hypersensitivity” was incorrectly abbreviated as Hhs with corresponding gene symbol *Hhs* throughout the abstract, main text, Methods, and figure and table captions. The trait and locus designations were updated by the Mouse Genome Informatics (MGI) database as Histh and *Histh*, respectively, prior to acceptance of the article but corrections were not carried out.

In addition, the following minor errors were identified:

- The caption to Table 2 incorrectly stated the amount of histamine injected as 1.0 mg. The correct dose was 50 mg/kg histamine.
- The following genetic cross was omitted from the Methods section “Animals”: (B10.S × SJL/J) × B10.S.
- The Methods section “Linkage analysis and generation of *Hhs* congenic mice” originally read: “Segregation of genotype frequency differences with susceptibility and resistance to Hhs in (B10.S × SJL/J) × B10.S were tested by Chi-square (*Χ*²) analysis in 2 × 2 contingency tables. B10.S-*Hhs*^SJL^ congenic mice were derived by marker-assisted selection of SJL/J derived alleles and successive backcrossing to B10.S mice.”
  The correct text should read: “Segregation of genotype frequency differences with susceptibility and resistance to Histh in (B10.S × SJL) F_2_ and (B10.S × SJL/J) × B10.S were tested by Chi-square (*Χ*²) analysis in 3 × 2 and 2 × 2 contingency tables, respectively. B10.S-*Histh*^SJL^ congenic mice were derived by marker-assisted selection of SJL/J derived alleles and successive backcrossing to B10.S mice.”
- The caption to Supplementary Data 1 table “Fixed” stated that 1.0 mg of histamine-free base was injected by i.v. in the experimental mice. The correct injection dose is 50 mg/kg.

The errors have now been corrected in both the PDF and HTML versions of the Article and in the Supplementary Data 1 file.

