# Supplementary material for: A natural mouse model reveals genetic determinants of systemic capillary leak syndrome (Clarkson disease)
Source: Commun Biol. 2019 Oct 31;2:398. doi: 10.1038/s42003-019-0647-4 (PMC6823437; doi:10.1038/s42003-019-0647-4)
Supplement: Supplementary file 1 — Description of Additional Supplementary Files [file 42003_2019_647_MOESM1_ESM.docx]

**Description of Additional Supplementary Files**

**File Name**: Supplementary Data 1

**Description**: Genetic linkage of mouse genome with histamine hypersensitivity (Hhs)

**File Name**: Supplementary Data 2

**Description**: Source data underlying the plots shown in Figures 1-6.

**File Name**: Supplementary Data 3

**Description**: Primer sequences used in this study.
