# Supplementary material for: A natural mouse model reveals genetic determinants of systemic capillary leak syndrome (Clarkson disease)
Source: Commun Biol. 2019 Oct 31;2:398. doi: 10.1038/s42003-019-0647-4 (PMC6823437; doi:10.1038/s42003-019-0647-4)
Supplement: Supplementary file 2 — Supplementary Information [file 42003_2019_647_MOESM2_ESM.pdf]

Supplementary Figure 1

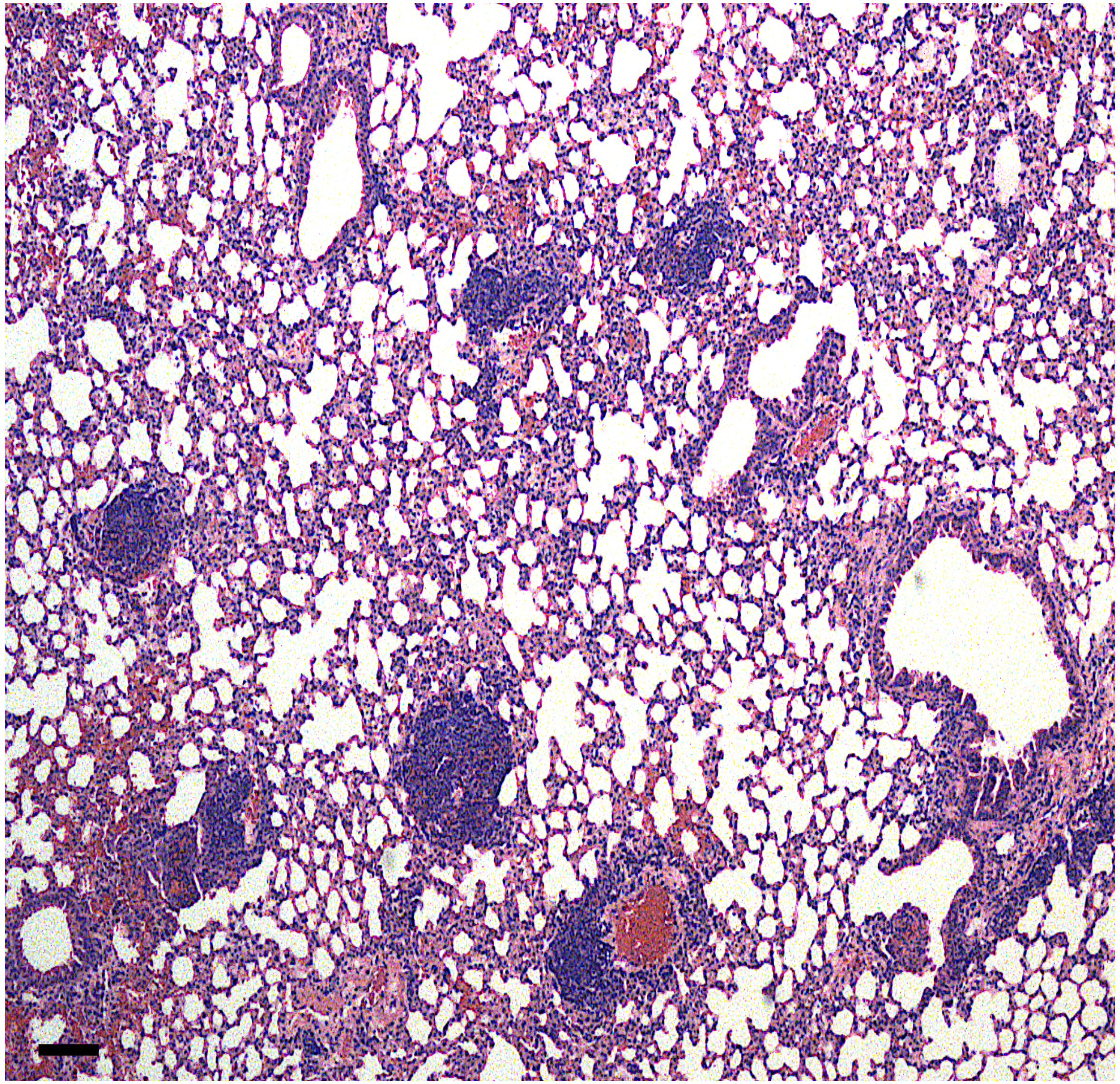

Supplementary Figure 1. Lung histology in untreated SJL mice. Lung tissue was obtained from aged (>six months of age) SJL mice. Tissue sections were stained with H&E and examined by microscopy. Image is from a single mouse representative of five mice. Scale bar=100 μm.
